# Meetings

**Published:** 1891-06

**Authors:** G. Munro Smith


### Bristol Medico-Chirurgical Society.

*April 8th*, 1891.

Mr. S. H. Swayne, President, in the Chair.

Dr. Harrison exhibited a man aged 67, who was the subject of lichen ruber. The disease appeared to follow a chill, and was first noticed as red spots on the chest, gradually spreading to the back and limbs: none came on the face, and very few on the neck. They varied from an eighth of an inch to an inch in diameter; the surface was smooth and shining; they were slightly raised above the skin-level, and were round, polygonal, or squarish. There was no history or suspicion of syphilis.

Dr. Prowse brought before the Society two cases of tumour of the pons (which will be published in full in the September number), and also the case of a young woman aged 19, who was admitted to the Infirmary on January 7th, 1891, and died on January 12th. There was a history of cyanosis and dyspnœa for some years; the legs had been œdematous for three months. The apex-beat was displaced to the left, and there was epigastric pulsation, presystolic bruit, and reduplicated first sound. Hæmoptysis supervened, and the dyspnœa became so urgent that it was decided to aspirate the right ventricle. This was accordingly done, and twelve ounces of blood withdrawn with great relief. A few hours later, however, the distress was as great as before, and death occurred rather suddenly. At the *post mortem* examination the mitral orifice was found greatly stenosed.

Mr. Pickering read notes of two cases of septic thrombosis of the lateral sinus. (These will be published in full in September.)

Dr. Edgeworth gave an account of the chief phenomena of hypnosis, and subsequently demonstrated these on a boy. He first explained the methods used to produce the hypnotic state, its various stages, and the alterations which take place (independently of suggestion) in voluntary movements, sensation and memory. He then stated the results of hypnotic and post-hypnotic suggestion on these faculties. These phenomena were illustrated on the subject. A discussion followed the demonstration.

---

*May 13th*, 1891.

Mr. S. H. Swayne, President, in the Chair.

Votes of thanks were passed to the British Medical Association and to R. B. Ruddock, Esq., for their generous and important gifts to the Library of the Society.

Dr. Watson Williams was requested and consented to act as delegate for the Society at the Congress for the Study of Tuberculosis, to be held in Paris in July next.

Dr. Michell Clarke read a short account of, and showed specimens from, three cases. (1) From a patient who died in the Hospital, under Mr. Dobson's care, from gangrene of the left foot and lower part of leg. She had left hemiplegia. There was no rheumatic history, and no murmur could be detected over the heart. At the *post mortem* examination, a large thrombus was found obstructing the abdominal aorta at its bifurcation. There were clots also in both common and external iliacs, and the right middle cerebral artery was blocked by an embolus; there were several infarcts in the left lung and kidney. The right lung was completely collapsed, apparently as a result of former pleuritic effusion. The mitral orifice only admitted the tip of the index-finger. The right cavities and left auricle were filled with clot. (2) Specimens from a case in which an old patch of softening had left a small cyst in the neighbourhood of the posterior part of the right internal capsule. This had caused left hemiplegia and hemianæsthesia. The symptoms pointed to vascular obstruction from thrombosis. The man died from a large fresh hemorrhage from the lenticulo-striate artery. He was the subject of Bright's disease. (3) Specimen of duodenal ulcer, the base of which was adherent to, and formed by, the gall-bladder. The latter contained cholesterin stones. There were no symptoms caused by the ulcer.

Dr. MARKHAM SKERRITT showed (1) Portion of a sarcoma of the right lung, which weighed 164 ounces, from a lad whose left thigh had been amputated for similar disease a year previously. The growth simulated some of the physical signs of extensive effusion, *e.g.* displacing heart, etc. It also pushed the diaphragm down three inches below the ribs, and could, therefore, be plainly felt in the left hypochondriac region. (2) A tumour of the right kidney, which microscopical examination showed to be an epithelioma with typical "nests" and penetrating columns of cells. The growth appeared to start from the neighbourhood of a calculus which obstructed the ureter about three inches below the pelvis. The symptoms had lasted five months.

Mr. C. A. MORTON showed (1) A specimen of syringo-myelocele. The cavity was directly continuous with the central canal of the cord above; below, the cord divided into two hollow parts. Nerves could be traced in the walls of the sac. Mr. Morton commented on the rarity of this condition of division of the cord. (2) The bones of an infant, the ends of which showed peculiar changes, probably syphilitic in nature. The epiphyses were separated.

Dr. GEORGE PARKER exhibited an aneurism, the size of a hazel-nut, situated on the basilar artery at its upper part. The case was remarkable from the complete absence of any localising symptoms. No cause for its occurrence was ascertained.

G. MUNRO SMITH, *Hon. Sec.*

---

